# Kisspeptin Receptor Agonists and Antagonists: Strategies for Discovery and Implications for Human Health and Disease

**DOI:** 10.3390/ijms26104890

**Published:** 2025-05-20

**Authors:** Xing Chen, Shu Yang, Natalie D. Shaw, Menghang Xia

**Affiliations:** 1 Division of Pre-Clinical Innovation, National Center for Advancing Translational Sciences (NCATS), National Institutes of Health (NIH), Rockville, MD 20850, USA; xing.chen@nih.gov (X.C.); shu.yang@nih.gov (S.Y.); 2 Pediatric Neuroendocrinology Group, Clinical Research Branch, National Institute of Environmental Health Sciences (NIEHS), National Institutes of Health (NIH), Research Triangle Park, NC 27709, USA; natalie.shaw@nih.gov

**Keywords:** kisspeptin, KISS1R, G protein-coupled receptor (GPCR), ligand, assay technology

## Abstract

The kisspeptin/kisspeptin receptor (*KISS1*/*KISS1R*) system has emerged as a vital regulator of various physiological processes, including cancer progression, metabolic function, and reproduction. *KISS1R*, a member of the G protein-coupled receptor family, is crucial for regulating the hypothalamic/pituitary/gonadal axis. A growing number of *KISS1R* agonists are currently being investigated in clinical trials, whereas the number of antagonists remains limited. Most existing ligands are synthetic peptides, with only a few small-molecule compounds, such as musk ambrette, having been identified. In this article, we provide an overview of the *KISS1*/*KISS1R* system and its involvement in diseases such as reproductive disorders, cancer, diabetes, and cardiovascular disease. We also highlight the various technologies used to identify *KISS1R* ligands, including radioligand binding assays, calcium flux assays, IP1 formation assays, ERK phosphorylation assays, qRT-PCR, and AI-based virtual screening. Furthermore, we discuss the latest advances in identifying *KISS1R* agonists and antagonists, highlighting ongoing challenges and future directions in research. These insights lay the groundwork for future research aimed at leveraging this system for developing innovative therapeutic strategies across a range of medical conditions.

## 1. Introduction

In recent decades, the kisspeptin/kisspeptin receptor (encoded by *KISS1*/*KISS1R*, respectively) system has attracted considerable research interest due to its role in diverse physiological processes [1,2,3]. Kisspeptin and its receptor are best known for their role in stimulating the reproductive axis, yet there has been a growing appreciation that kisspeptin signaling affects an extremely diverse set of biological processes, including ovarian function, placentation, angiogenesis, metabolism, kidney development, olfaction, sexual behavior, and mood [4,5]. Despite this emerging collection of data, research into endogenous and exogenous ligands targeting *KISS1R* remains quite limited [6,7,8,9,10,11]. This review, therefore, aims to summarize what is known about kisspeptin receptor ligands, their roles in human physiology, and potential therapeutic applications for kisspeptin agonists and antagonists to treat human disease.

## 2. The Kisspeptin/Kisspeptin Receptor System

Kisspeptin, the *KISS1* gene product, was first discovered to be a metastasis suppressor in a screen of human malignant melanoma cells [12], and soon thereafter, was also found to suppress breast cancer metastases [13], leading to its initial designation as ‘Metastin’ [14]. Surprisingly, studies reported very low *KISS1* expression in tumor cells, whereas the highest expression was seen in the human placenta, followed by the pancreas, liver, and small intestine [12,14]. Kisspeptin increases several thousand-fold in the maternal circulation during human pregnancy, with the placenta believed to be its primary source. During pregnancy, kisspeptin plays important roles in embryo implantation, placentation, and maternal glucose homeostasis [15].

Although these early studies did not identify strong *KISS1* expression in whole-brain samples, two seminal studies in 2003 demonstrated that kisspeptin signaling in the hypothalamus plays a critical role in the neuro-endocrine control of puberty and reproduction: Seminara et al. [16] and de Roux et al. [17] discovered that loss-of-function mutations in *GPR54* (also known as *KISS1R*) cause congenital gonadotropin-releasing hormone (GnRH) deficiency in humans and mice. Conversely, gain-of-function mutations in *KISS1* and *KISS1R* cause precocious (early) puberty [18,19]. In humans, kisspeptin is expressed in hypothalamic KNDy neurons (so-called because they secrete three neuropeptides, kisspeptin, neurokinin B, and dynorphin), which project to and strongly stimulate GnRH neurons to release GnRH. GnRH then stimulates the pituitary gland to secrete luteinizing hormone (LH) and follicle-stimulating hormone (FSH), two hormones that modulate testicular and ovarian function. Importantly, KNDy neurons express receptors for sex steroids, leptin, insulin, and glucocorticoids, suggesting that they serve as a central hub in sensing and integrating internal and external cues to promote or suppress reproductive axis activity (e.g., when energy is scarce) [20].

**Figure 1:**
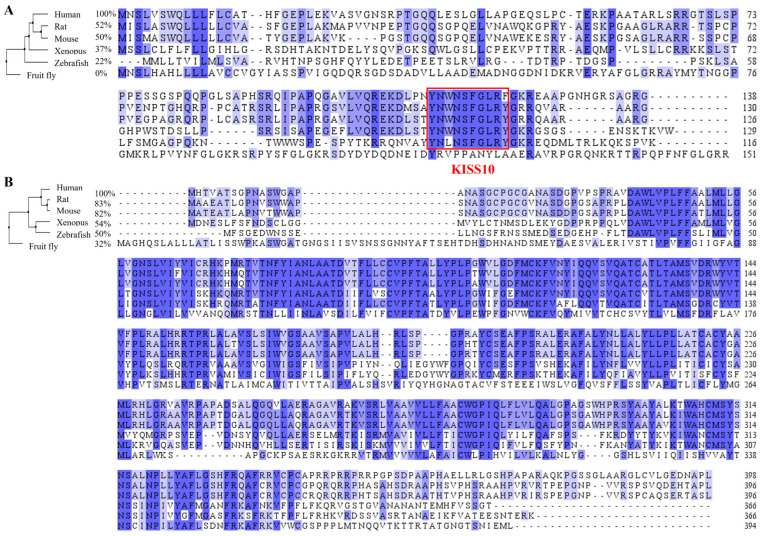
Alignment of amino acid sequences of *KISS1*/*KISS1R* protein from humans and five other species. (**A**) *KISS1* protein sequence alignment. The sources of sequences are human (GenBank, NP_002247.3), rat (*Rattus norvegicus*) (GenBank, NP_859043.1), mouse (*Mus musculus*) (GenBank, NP_839991.2), Xenopus (*Xenopus tropicalis*) (GenBank, NP_001156331.1), zebrafish (*Danio rerio*) (GenBank, NP_001106961.1), and fruit fly (*Drosophila melanogaster*) (GenBank, NP_524489.2). The red box is the KP-10 amino acid area. (**B**) *KISS1R* protein sequence alignment. The sources of sequences are human (GenBank, NP_115940.2), rat (GenBank, NP_076482.2), mouse (GenBank, NP_444474.1), Xenopus (GenBank, NP_001163985.1), zebrafish (GenBank, NP_001099149.2), and fruit fly (GenBank, NP_524700.1). Jalview software (version 2.11.3.0) was used for alignment and coloring with Percentage Identity. The intensity of the purple shade indicates the percentage abundance of aligned amino acids. The percentage identities with the human *KISS1*/*KISS1R* amino acid sequence were calculated based on pairwise alignment in Jalview.

The human *KISS1* gene encodes a 138-amino-acid pre-proprotein that undergoes proteolytic cleavage to a number of bioactive kisspeptins, including kisspeptin 54 (KP-54), kisspeptin 14 (KP-14), kisspeptin 13 (KP-13), and kisspeptin 10 (KP-10). All of these proteins act as agonists of *KISS1R* [1,14]. Interspecies conservation of the kisspeptin amino acid sequences is limited, even among mammalian species, with only 50–52% homology observed between human kisspeptin and that of rodents and even greater divergence from nonmammalian sequences (Figure 1A). Despite this overall divergence, the amino acid sequence of KP-10, the shortest peptide capable of activating *KISS1R* [14], displays high conservation across species, exceeding 80% similarity in most cases (Figure 1A). While human KP-10 terminates with an Arg-Phe-NH2 motif (RFamide), other species, such as zebrafish [21], rats [22], mice [22], and *Xenopus* [21], exhibit a RYamide at the C-terminus.

Notably, *KISS1R* is highly conserved across vertebrates (Figure 1B), highlighting the fundamental nature of signaling pathways mediated by *KISS1R* in vertebrate reproductive physiology. *KISS1R* predominantly signals through the Gq-protein/phospholipase C/inositol (1,4,5)-triphosphate (IP3) pathway, leading to intracellular calcium release [23,24]. Additional pathways include protein kinase C activation, extracellular signal-regulated kinase (ERK) phosphorylation, and phosphatidylinositol-3 kinase (PI3K)/Akt [24,25]. Recent studies have revealed that *KISS1R* activation results in increased intracellular cAMP levels [26,27], suggesting crosstalk between Gq- and Gs-protein signaling pathways. Alterations in downstream molecules within the kisspeptin pathway, such as intracellular calcium flux, the formation of D-myo-inositol monophosphates (IP1) (an IP3 degradation product), and phosphorylated ERK (p-ERK), are frequently employed as targets when identifying potential *KISS1R* agonists and antagonists [25].

## 3. Role of the Kisspeptin System in Human Disease

### 3.1. Reproductive Disorders

**Figure 2:**
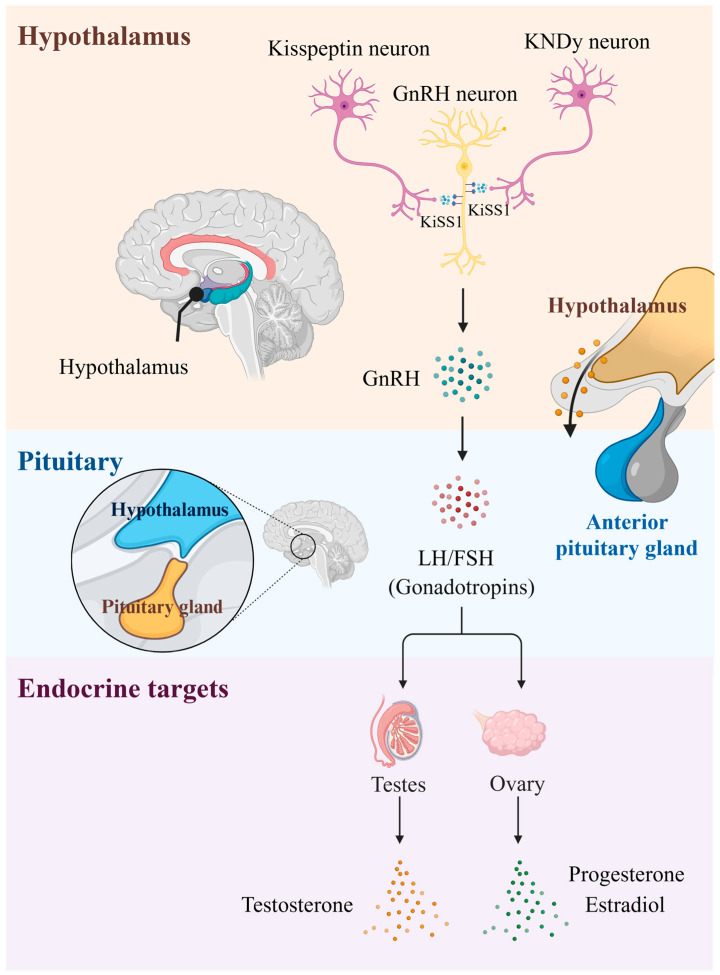
Kisspeptin and the hypothalamic/pituitary/gonadal (HPG) axis. Arrows in the diagram denote the direction of hormone transport. GnRH: gonadotropin-releasing hormone; KNDy: kisspeptin/neurokinin B/dynorphin; LH: luteinizing hormone; FSH: follicle-stimulating hormone. The graph was created with BioRender. Yang, S. and Chen, X. (2025), https://app.biorender.com/illustrations/682571c1cba59c4817a70d80 (accessed on 8 April 2025).

Kisspeptin’s interaction with GnRH neurons is pivotal for reproductive function. The colocalization of *KISS1R* and *GnRH* expression was first demonstrated in a tilapia model system, indicating that GnRH neurons are direct targets of kisspeptin [28,29]. Subsequent studies employing double-label in situ hybridization revealed that over 75% of rat GnRH neurons co-express *KISS1R* mRNA, further confirming this direct interaction [30]. The signaling cascade elicited by *KISS1R* activation stimulates GnRH secretion, which then stimulates the release of pituitary LH and FSH, which drive gonadal sex steroid secretion [29,31]. This process is depicted in Figure 2. As noted previously, mutations in *KISS1*/*KISS1R* cause congenital GnRH deficiency (characterized by delayed or absent puberty and infertility) [17] or central precocious puberty [18], and aberrant kisspeptin signaling has been implicated in polycystic ovary syndrome (PCOS) [32] and hypothalamic amenorrhea [33].

### 3.2. Cancer

Increased *KISS1* mRNA expression is associated with reduced metastatic potential, potentially through the inhibition of tumor cell migration and invasion [6]. Early expression studies by Lee et al. in a panel of human melanoma cells demonstrated that *KISS1* expression occurred only in nonmetastatic melanoma cells [12]. Further studies revealed that the suppression of *KISS1*/*KISS1R* signaling allows for the progression and metastasis of osteosarcoma, gastric, prostate, and breast cancer [6,34]. While kisspeptin signaling has shown tumor-suppressive effects in colorectal cancers [35], findings in triple-negative breast cancer suggest pro-tumorigenic roles under certain conditions [36]. For instance, the short hairpin RNA-mediated knockdown of *KISS1R* in MDA-MB-231 breast cancer cells reduces invadopodia formation and invasive behavior via the disruption of β-arrestin2/ERK1/2 signaling [36]. In contrast, *KISS1R* knockdown in colorectal cancer cells leads to increased migration and invasion, reinforcing its tumor-suppressive potential in that context [35]. Complementing these in vitro observations, heterozygous *Kiss1r*^+^/^−^ mice in a breast cancer model exhibited reduced breast tumor initiation and lung metastasis [37]. These observations support the context-dependent role of the *KISS1*/*KISS1R* axis in cancer. Nonetheless, the possibility of off-target effects in non-receptor-specific models remains a valid consideration and underscores the need for mechanistic studies using well-validated, receptor-selective tools. For example, other G protein-coupled receptors (GPCRs) implicated in cancer metastasis, such as CXC receptor 4 (Gi-coupled) and protease-activated receptor-1 (Gq-coupled), often activate similar mitogen-activated protein kinase (MAPK) and PI3 kinase/protein kinase B (PI3K/AKT) pathways and may also be involved in the observed cellular phenotypes [38,39,40].

### 3.3. Diabetes and Metabolism

Recent studies suggest that the kisspeptin system plays a significant role in insulin secretion and glucose homeostasis. Both *KISS1* and *KISS1R* mRNA are expressed in pancreatic islets, and kisspeptin stimulates glucose-induced insulin secretion in both humans and mice [41] via a phospholipase C-dependent pathway that increases intracellular calcium, as demonstrated in mouse islets and rat models [42]. This mechanism distinguishes it from incretin receptors, like the glucagon-like peptide-1 (GLP-1) receptor, which enhances insulin and inhibits glucagon secretion through Gs to increase cyclic AMP (cAMP) and activate protein kinase A (PAK) [43].

The regulatory actions of kisspeptin on glucose homeostasis involve a liver-to-pancreatic islet endocrine circuit [44]. Glucagon stimulates hepatic *KISS1* expression through the cAMP-PKA- response element binding protein (CREB) pathway, leading to increased circulating kisspeptin. This liver-derived kisspeptin, in turn, suppresses glucose-stimulated insulin secretion from pancreatic β-cells [44]. Kisspeptin levels are increased in adults with type 2 diabetes mellitus and in mouse models of diabetes [44,45], and global *KISS1R* knockout [46] and knockdown of liver *KISS1* in particular improve glucose tolerance [44].

### 3.4. Cardiovascular Disease

Recent research has revealed the significant role of the kisspeptin system in cardiovascular disease, particularly in vascular tone regulation, atherosclerosis, and cardiac remodeling. Kisspeptins have been identified as potent vasoconstrictors in human blood vessels, with their receptor *KISS1R* being selectively localized to atherosclerosis-prone vessels such as the aorta and coronary arteries [47]. This suggests that kisspeptin signaling may contribute to vascular dysfunction and the progression of atherosclerosis. Additionally, KP-10 has been implicated in plaque instability by promoting smooth muscle contraction within atherosclerotic plaques, potentially increasing the risk of rupture and acute cardiovascular events [48]. KP-10 also enhances collagen deposition in the myocardium, leading to fibrosis and adverse cardiac remodeling [49]. However, research studies on humans have found no significant changes in blood pressure or heart rate following KP-54 administration [50]. Taken together, these findings suggest that the kisspeptin system may play a role in cardiovascular health, warranting further investigation into its therapeutic potential.

## 4. Assay Technologies to Identify *KISS1R* Ligands in a High-Throughput Format

As the dysregulation of the kisspeptin system has been linked to a number of human diseases, the development of *KISS1R* agonists and antagonists may help fill an unmet need for therapeutics in multiple disciplines. *KISS1R* belongs to the GPCR family, a major focus of pharmaceutical drug development. Numerous well-established high-throughput screen (HTS) assay methods have already been developed and refined for GPCRs [51,52]. In addition, we [25] and others [53] have configured HTS assays to measure a compound’s ability to activate *KISS1R*, relying on inositol phosphate formation, calcium flux, ERK phosphorylation, and GnRH gene transcription, for example. In this review, we will introduce the key targets and corresponding detection assays suitable for screening *KISS1R* ligands in a high-throughput format (384-well or 1536-well plate). These same principles can be applied to other GPCRs.

### 4.1. Radioligand Binding Assay

Radioligand binding assays can be used to identify *KISS1R* ligands, providing quantitative data on ligand/receptor interactions. These assays utilize radiolabeled kisspeptins, such as [125I]-KP-10, to measure their binding affinity to the receptor [1,54,55]. By incubating radiolabeled ligands with receptor-expressing cells or membrane preparations and assessing their binding in the presence or absence of unlabeled competitors, researchers can determine key parameters like ligand affinity (Kd) and potency (IC_50_). This approach enables the identification of novel ligands, including both endogenous peptides and synthetic compounds. However, limitations exist, including the potential for non-specific binding and the fact that binding assays may not always directly reflect physiological receptor activity [56]. Radioligand binding assays can be miniaturized and automated, utilizing techniques like filter-based assays or scintillation proximity assays in 96- or 384-well plates, enabling the rapid screening of large compound libraries [57].

### 4.2. Calcium Flux Assay

The activation of *KISS1R* leads to intracellular Ca^2+^ release from the endoplasmic reticulum. This process is quantifiable using calcium-sensitive dyes such as Fluo-4 and Cal-520, in conjunction with rapid-injection imaging platforms [58,59]. However, many fluorescent compounds, calcium channel-regulating compounds, and toxicants can also trigger intracellular calcium release independently from the *KISS1R*, leading to false-positive results [59]. To mitigate these effects, studies often utilize wild-type cells (e.g., that do not overexpress *KISS1R*) or *KISS1R* knockout cell lines as controls to remove these false positives. Candidate compounds can also be validated by investigating how compounds interact with endogenously expressed receptors, such as by utilizing hypothalamic neurons in the case of *KISS1R*. The potential drawbacks of this approach include the possibility that genetic modifications used to knock in or knock out the receptor of interest can induce compensatory changes in gene expression, potentially confounding data interpretation. The high cost of rapid-injection imaging platforms also limits their widespread use in screening studies.

### 4.3. IP1 Formation Assay

An alternative approach for detecting *KISS1R* activation is to measure inositol phosphate (IP) formation. However, the rapid degradation of IP3 poses a challenge for direct detection [60]. Instead, an IP1 formation assay was developed, leveraging the ability of lithium chloride (LiCl) to inhibit the degradation of IP1, leading to its accumulation in the cell [61]. Using homogeneous time-resolved fluorescence (HTRF) detection technology, this assay can be performed in high-throughput formats without requiring wash steps [60].

**Figure 3:**
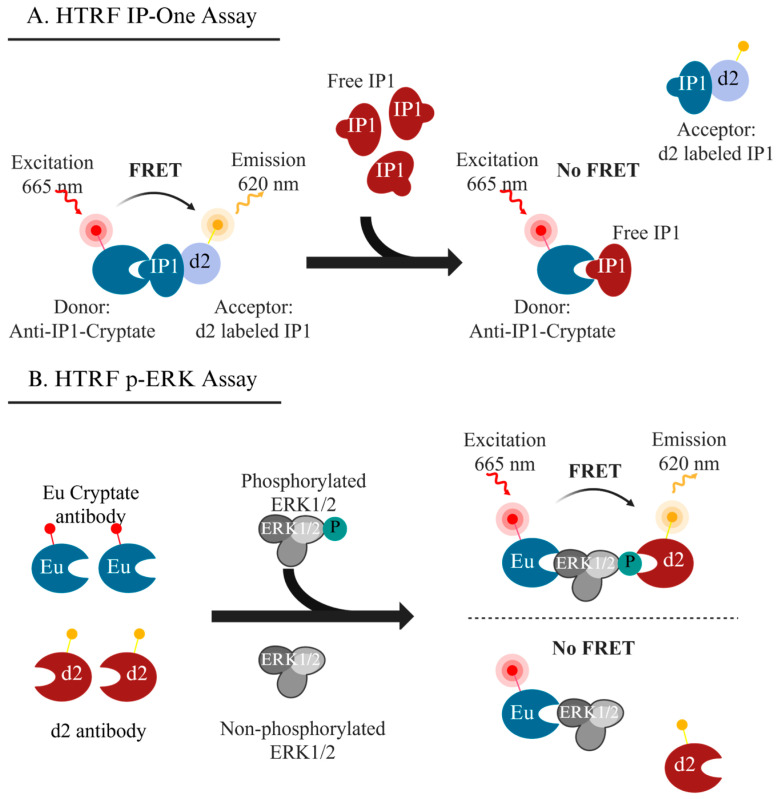
The principle of the homogeneous time-resolved fluorescence (HTRF) assay. (**A**) HTRF used in detection of IP1; (**B**) HTRF used in detection of phosphorylated ERK (p-ERK). IP1: D-myo-inositol monophosphates; FRET: fluorescence resonance energy transfer. The graphs were created with BioRender, Chen, X. (2025), https://app.biorender.com/illustrations/682571c1cba59c4817a70d80 (accessed on 8 April 2025).

In the HTRF assay, an antibody binds to d2-labeled IP1, bringing it into proximity with a donor fluorophore (Tb cryptate). This proximity enables the transfer of energy from a donor fluorophore (Tb cryptate) to an acceptor fluorophore (d2), resulting in fluorescence emission [62,63,64] (Figure 3A). While this competitive immunoassay offers valuable specificity, it is contingent upon the availability of the anti-IP1 antibody. Any competing proteins that can bind this antibody may yield false positives. Additionally, the assay’s sensitivity could be compromised by weak *KISS1R* agonists that do not generate sufficient IP1 to compete with d2 fluorophore-labeled IP1, potentially leading to undetectable or false-negative results. Furthermore, since IP1 is not a direct product of *KISS1R* activation and requires the prior formation of IP3, compounds affecting the degradation of IP3 to IP1 may also skew assay results. Consequently, careful consideration of assay development and validation is essential for researchers utilizing this method.

### 4.4. Assays Targeting ERK Phosphorylation

The phosphorylation of ERK (p-ERK) can also be monitored to detect *KISS1R* activity [25,26,65,66]. For HTS purposes, both HTRF and AlphaLISA (amplified luminescence proximity homogenous assay) can effectively detect p-ERK. These immunoassays utilize two antibodies targeting the same biomolecule, facilitating energy transfer (in HTRF) (Figure 3B) or singlet oxygen generation (in AlphaLISA) to produce a fluorescence or a luminescent signal, respectively [67,68].

Despite providing high sensitivity and low background noise, these assays still require optimization to determine peak detection times for p-ERK following treatment. As ERK phosphorylation is not exclusively linked to *KISS1R* activation, other compounds that activate alternative receptors or pathways may also induce p-ERK, confounding results. Consequently, it is advisable to combine these assays with others to comprehensively evaluate *KISS1R* ligands, even when employing *KISS1R*-overexpressing cell lines.

### 4.5. qRT-PCR to Detect GnRH Expression

In human hypothalamic cell lines, *GnRH* expression is a validated marker of *KISS1R* activation. However, *GnRH* is not a downstream target in all cells that express *KISS1R*. The advent of high-throughput one-step quantitative reverse transcription polymerase chain reaction (qRT-PCR) technology enables the assessment of gene expression across 384-well plates. However, the lack of PCR equipment for 1536-well plate formats presents a limitation on further increasing the throughput.

### 4.6. Potential AI-Based Virtual Screening Technology

In the last decade, artificial intelligence (AI) technologies have increasingly entered the drug discovery pipeline, facilitating the identification of promising compounds for further development. AI-based programs such as PyRMD [69] and RosettaGenFF-VS [70] utilize machine learning algorithms and computational models to predict the binding affinity and bioactivity of chemical compounds prior to experimental validation, dramatically enhancing the efficiency of ligand identification for targets such as *KISS1R*. An advantage of AI-based virtual screening lies in its capability to efficiently process large chemical libraries, significantly reducing the time and costs associated with traditional high-throughput screening methods.

By analyzing complex molecular interactions and optimizing lead compounds based on predictive models, AI technologies enable the identification of potential drug candidates that might be overlooked using conventional methods. Moreover, AI can refine specificity in compound selection by integrating diverse biological, chemical, and pharmacological data, leading to more targeted ligand identification. However, challenges remain, primarily due to the dependence on the quality and diversity of input data, which can influence model accuracy and predictive capacity.

Recent structural studies have significantly advanced our understanding of how *KISS1R* recognizes its ligands and couples with specific G proteins. Wu et al. (2024) demonstrated *KISS1R*’s capacity for coupling to both the Gq/11 and Gi/o pathways [71]. Their cryo-EM structures of *KISS1R*-Gq/Gi complexed with KP-54 or TAK-448 (a kisspeptin receptor agonist) provided key insights into both ligand recognition and G protein selectivity [71]. Shen et al. (2024) reported another high-resolution *KISS1R* structure, elucidating ligand binding features with KP-10 and TAK-448 at its extracellular loops [72]. Their analysis identified a unique 40° angular deviation in the intracellular TM6 region, essential for distinct Gq interactions [72]. These findings have the potential to facilitate AI-based screening for novel *KISS1R* ligands, an approach that has been successfully applied to other GPCRs [73]. Experimental validation remains crucial, as computational models may not fully account for the dynamic biological environments in which these interactions occur. Moreover, docking predictions typically struggle to effectively distinguish between agonists and antagonists from ligand binding data, necessitating experimental confirmation. As such, AI-based virtual screening technology offers substantial advantages within *KISS1R* research, but hybrid methodologies that combine computational and experimental approaches are essential for validating findings.

## 5. Ligands of *KISS1R*

The exploration and development of ligands targeting *KISS1R* are paramount for discovering innovative therapeutics in cancer, reproductive medicine, and metabolic disorders. Currently, researchers are investigating both agonists and antagonists of *KISS1R* as potential treatments for reproductive disorders, such as infertility, hypogonadism, and PCOS [74].

### 5.1. Agonists

Both natural and synthetic compounds have been identified as effective agonists that activate the kisspeptin receptor. This activation results in stimulating critical signaling pathways involved in cancer progression, metabolic regulation, and reproductive physiology. Various in vitro and in vivo assays have been developed to assess *KISS1R* ligand binding affinity, the kinetics of receptor activation, and the molecular function of agonists [75]. A summary of the agonists identified to date is provided in Table 1. Notably, all of these kisspeptin receptor agonists are natural or synthetic peptides with the exception of musk ambrette.

#### 5.1.1. Kisspeptins (KP-54, KP-14, KP-13, KP-10)

KP-54, KP-14, KP-13, and KP-10 are endogenous ligands of *KISS1R* that are derived from the kisspeptin pre-proprotein. They comprise 54, 14, 13, and 10 amino acids, respectively [1]. Binding assays reveal that they exhibit the same affinity and efficacy at the kisspeptin receptor despite their different lengths [1].

KP-54 and KP-10 have been investigated in clinical trials for treating metabolic or reproductive disorders such as impaired glucose tolerance, hypogonadotropic hypogonadism, hyperprolactinemia, and delayed puberty (ClinicalTrials.gov ID: NCT02953834, NCT04648969, NCT02956447, NCT081924, NCT04532801, NCT05633966, NCT05896293, NCT00914823, NCT01438034, NCT05456854, NCT03771326, NCT04975347, NCT01952782, NCT01438073, NCT03286517, and NCT01667406) [92]. However, the limited publications reporting these findings [93,94,95,96] underscore the significant amount of work required in this research field.

#### 5.1.2. FTM080

**Figure 4:**
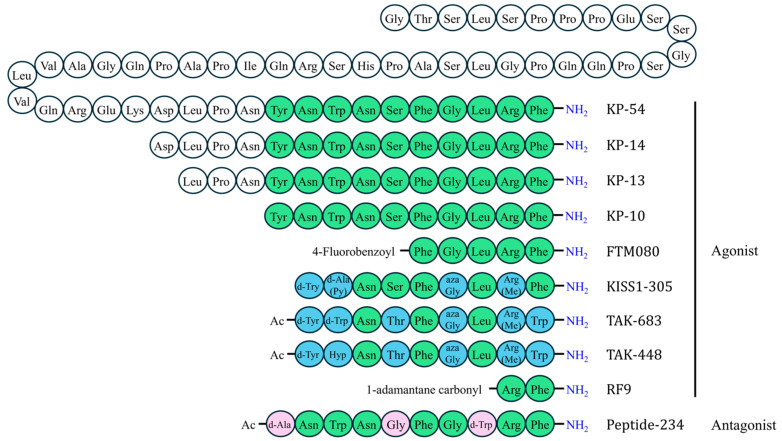
Amino acid sequences of kisspeptin 10 (KP-10) analogs. Amino acid residues are color-coded to indicate their relationship to native KP-10: green for identical residues, blue for agonist analog substitutions, pink for antagonist analog substitutions, and white for additional amino acids.

A variety of peptide analogs (compounds replicating peptide structure and function) of KP-10 have been developed, such as FTM080, *KISS1*-305, TAK-448 (MVT-602), and TAK-683 [55,78,79,84]. All of these are synthetic kisspeptin analogs but differ in their specific protein sequences. *KISS1*-305, TAK-448, and TAK-683 are nonapeptides, and FTM080 is a pentapeptide. *KISS1*-305 ends with Arg-Phe-NH_2_, identical to KP-10, whereas FTM080, TAK-448, and TAK-683 end with Arg-Trp-NH_2_. The analog peptide structures are illustrated in Figure 4.

FTM080, identified as 4-Fluorobenzoyl-Phe-Gly-Leu-Arg-Trp-NH_2_, is notable for its increased resistance to enzymatic degradation, particularly from matrix metalloproteinases (MMPs), compared to KP-10 [79]. The objective of structural modifications, especially in the C-terminal region, is to enhance stability and half-life in the bloodstream, enabling the peptides to maintain prolonged functional activity [80]. In vitro studies indicate that FTM080 exhibits binding affinity and efficacy comparable to KP-10 against human *KISS1R* [78,79]. However, promising in vitro results have been tempered by conflicting follow-up in vivo studies in sheep, indicating that the biological responses may not correlate directly with in vitro performance. The potential to stimulate the gonadotropic axis was demonstrated by a seven-fold increase in plasma LH concentrations due to the intravenous administration of FTM080 in animal studies [80].

#### 5.1.3. *KISS1*-305

Asami et al. discovered that amino acid substitutions at positions 45–47 of kisspeptin enhanced both the agonistic activity at *KISS1R* and protein stability, and N-terminal truncation further improved stability [55]. Based on these findings, a nonapeptide analog of KP-10, *KISS1*-305, was rationally designed to resist degradation caused by plasma proteases while retaining agonistic activity at *KISS1R* [55,81] (Figure 4).

Comparable to KP-10, *KISS1*-305 exhibits robust agonistic activity in vitro, with a half-maximal effective concentration (EC_50_) of 4.8 nM [55]. Significant increases in plasma LH and testosterone levels have also been observed in male rodents following the acute administration of *KISS1*-305 [81]. *KISS1*-305 has been reported to be more appropriate for therapeutic application due to its protease resistance [81].

#### 5.1.4. TAK-683

TAK-683 was synthesized to enhance stability and address the deficiencies of *KISS1*-305 at physiological pH, which includes physicochemical instability and the weakness of in vitro activity [87]. Key modifications included the substitutions of N-terminal acetyl, d-Trp^47^, and Trp^54^ in TAK-683, which contribute to improved stability and *KISS1R* agonistic potency [87]. The agonistic activities of TAK-683 have been well documented [87], and its peptide sequence is illustrated in Figure 3.

TAK-683 has undergone investigations for safety, tolerability, pharmacokinetics, and pharmacodynamics: in two randomized, double-blind, placebo-controlled studies, Scott et al. demonstrated that a single dose of TAK-683 caused an increase in LH, FSH, and testosterone levels in healthy men [86]. TAK-683 proved to be well-tolerated at all administered concentrations.

#### 5.1.5. TAK-448 (MVT-602)

TAK-448, also known as MVT-602, was designed and synthesized by Takeda Pharmaceutical Company [84]. TAK-448 was developed as an optimized version of TAK-683, addressing specific limitations such as gel formation [84]. Both TAK-448 and TAK-683 are nonapeptides displaying high receptor-binding affinity, with full agonistic activity for rat *KISS1R*, akin to the effects of KP-10 [87] (Table 1). Notably, the primary difference between TAK-448 and TAK-683 lies in the substitution of d-Trp at position 47 with trans-4-hydroxyproline (Hyp) (Figure 4).

Clinical and in vitro studies have also indicated that TAK-448 may serve as a therapeutic option for reproductive disorders [85,97]. In a study involving nine healthy women, six women with PCOS, and six women with hypothalamic amenorrhea, TAK-448 induced a more sustained increase in LH levels than KP-54, particularly in the latter group. TAK-448 induced more potent *KISS1R* activation (as determined by measuring IP1 accumulation) and a longer duration for GnRH neuronal firing than KP-54. Thus, TAK-488 holds the potential to treat reproductive disorders, and clinical trials are ongoing (ClinicalTrials.gov ID: NCT02381288, NCT02369796, NCT01132404).

#### 5.1.6. RF9 (1-Adamantane Carbonyl-Arg-Phe-NH2)

RF9 was initially reported to be a potent and selective antagonist of neuropeptide FF receptor (NPFFR) and was employed to prevent opioid-induced hyperalgesia [98]. Subsequent studies discovered that RF9 increased gonadotropin secretion in rats and sheep [88,89] and that RF9 excites GnRH neurons in a *KISS1R*-dependent manner [90]. In Chinese hamster ovary cells stably transfected with human *KISS1R*, RF9 was shown to bind to *KISS1R* with a dissociation constant (Kd) of 1.6 × 10^−5^ M and to stimulate intracellular calcium release and inositol phosphate accumulation in a *KISS1R*-dependent manner, with EC_50_ values of 3.0 × 10^−6^ M and 1.6 × 10^−7^ M, respectively [91]. RF9 also triggered an LH surge in wild-type mice but not in *Kiss1r* knockout mice [91]. These findings indicate that RF9 acts as a *KISS1R* agonist both in vitro and in vivo.

#### 5.1.7. Musk Ambrette

We recently conducted a quantitative high-throughput screening of the Tox21 10K compound library to identify small-molecule agonists of human *KISS1R* [25]. Human *KISS1R* was interrogated using HEK293 cell lines overexpressing *KISS1R*. A selected set of candidate agonists underwent further investigation, including Ca^2+^ flux and ERK phosphorylation assays. These data pointed to musk ambrette, 2,6-dinitro-3-methoxy-4-tert-butyltoluene, as a novel *KISS1R* agonist. Further investigation confirmed that musk ambrette increases *Gnrh* expression in mouse and human hypothalamic cells, as well as in transgenic *gnrh3:gfp* zebrafish. Musk ambrette is a synthetic nitro musk compound characterized by a substituted benzene ring bearing two nitro groups, a methoxy group, a tert-butyl group, and a methyl group [99]. It has a molecular weight of 269.28 g/mol. Historically, musk ambrette was widely used as a fragrance ingredient in perfumes, soaps, and cosmetics [99]. However, its use has declined due to concerns about its toxicity and environmental persistence [99,100].

### 5.2. Antagonists

To evaluate the binding affinity and molecular function of antagonists, the same assay methodologies used for agonist identification are typically applied. These methods include radioligand binding assays, fluorescence-based assays, and functional assays that measure intracellular signaling responses. A summary of the reported antagonists is presented in Table 2.

#### 5.2.1. Peptide-234

Peptide-234 is a modified peptide derived from the natural kisspeptin ligand KP-10. The peptide is altered by substituting D-Trp for Leu^8^, Gly for Ser^5^, and D-Ala for Tyr^1^ [101]. These modifications confer antagonist activity to peptide-234 [101]. Peptide-234 inhibits GnRH neuronal firing in mice and reduces GnRH/LH pulses in rhesus monkeys and ovariectomized sheep [101]. When administered to pubertal female rats, peptide-234 also delayed vaginal opening, decreased uterine and ovarian weights, prevented preovulatory LH and FSH surges, and blunted the rise in gonadotropins in response to KP-10 [102].

#### 5.2.2. 2-Acylamino-4,6-Diphenylpyridine Derivatives

Kobayashi et al. conducted a high-throughput screening of the Takeda proprietary compound collection to identify a *KISS1R* antagonist. Further analyses of the hit compound and related 2-acylamino-4,6-diphenylpyridine derivatives led to the identification of compound 9l, the most potent antagonist, with an IC_50_ of 3.7 nM in human *KISS1R* assays [103]. In vivo studies confirmed its efficacy in reducing plasma LH levels in castrated male rats [104].

**Table 2:** Activities of *KISS1R* antagonists.

Compound	Object	Concentration	Measurement	Result	Reference
Peptide-234	CHO cells expressing human *KISS1R*	10 pM–10 μM	Whole-cell receptor binding assay	IC_50_ = 2.7 nM	Roseweir et al., 2009 [101]
	CHO cells expressing human *KISS1R*	100 pM–1 μM	IP1	IC_50_ = 7 nM	Roseweir et al., 2009 [101]
	Female *GnRH*–GFP mice	1–100 nM	Targeted extracellular recording	Blocked GnRH neuron firing by 1 nM KP-10	Roseweir et al., 2009 [101]
	Female rhesus monkey	10 nM	GnRH level	Inhibited pulsatile GnRH release	Roseweir et al., 2009 [101]
	Male rats and mice	1/15 nM	LH level	Inhibited KP-10 stimulated LH	Roseweir et al., 2009 [101]
	Ovariectomized ewe	40 μg	LH level	Inhibited LH secretory pulse	Roseweir et al., 2009 [101]
	Ovariectomized rats	10/50 pM	LH level	Inhibited LH secretory pulse	Li et al., 2009 [105]
2-acylamino-4,6-diphenylpyridine	CHO cells expressing human *KISS1R*	NA	Binding assay	IC_50_ = 1.5 µM	Kobayashi et al., 2010 [103]
	CHO cells expressing human *KISS1R*	10 μM	Ca^2+^ assay	58% inhibition	Kobayashi et al., 2010 [103]
2-acylamino-4,6-diphenylpyridine derivative, 9l	CHO cells expressing human *KISS1R*	NA	Binding assay	IC_50_ = 3.7 nM	Kobayashi et al., 2010 [103]
	CHO cells expressing human *KISS1R*	NA	Ca^2+^ assay	IC_50_ = 0.46 µM	Kobayashi et al., 2010 [103]
2-acylamino-4,6-diphenylpyridine derivative, 15a	CHO cells expressing human *KISS1R*	NA	Binding assay	IC_50_ = 3.6 nM	Kobayashi et al., 2010 [104]
	CHO cells expressing human *KISS1R*	1 nM–100 μM	Ca^2+^ assay	IC_50_ = 0.93 µM	Kobayashi et al., 2010 [104]
	Castrated male rats	0.22 mg/kg	LH level	Reduced plasma LH level	Kobayashi et al., 2010 [104]

NA, not available.

#### 5.2.3. Other Studies

In 2010, Kuohung et al. screened the small-molecule library of the Laboratory for Drug Discovery in Neurodegeneration (LDDN) at Brigham and Women’s Hospital (Boston, MA, USA) to identify small-molecule antagonists (and agonists) of *KISS1R* [53]. To the best of our knowledge, the antagonists have not undergone further characterization.

## 6. Future Perspective

In this review, we have highlighted the importance of the *KISS1*/*KISS1R* system in human biology, outlined strategies for identifying ligands that interact with GPCRs, and reviewed progress in discovering and/or rationally designing *KISS1R* agonists and antagonists. As this field continues to grow, new therapies are likely to be unveiled that will impact the fields of reproductive medicine, oncology, and metabolism.

## Figures and Tables

**Table 1:** Activities of *KISS1R* agonists.

Compound	Object	Concentration	Measurement	Result	Reference
KP-54	CHO-K1 cells expressing human or rat *KISS1R*	10 pM–1 µM	Competitive binding assay	Human *KISS1R*: K_i_ = 1.45 ± 0.1 nM; rat *KISS1R*: K_i_ = 1.81 ± 0.05 nM	Kotani et al., 2001 [1]
	CHO-K1 cells expressing human or rat *KISS1R*	10 pM–1 µM	Aequorin-based functional assay to measure Ca^2+^	Human *KISS1R*: EC_50_ = 5.47 ± 0.03 nM; rat *KISS1R*: EC_50_ = 1.39 ± 0.03 nM	Kotani et al., 2001 [1]
	Women	0.2–6.4 nmol/kg	LH level	Increased	Dhillo et al., 2007 [76]
	Women	1.6–12.8 nmol/kg	Egg maturation	Increased mature egg number	Jayasena et al., 2014 [32]
KP-14 [1]	CHO-K1 cells expressing human or rat *KISS1R*	10 pM–1 µM	Competitive binding assay	Human *KISS1R*: K_i_ = 1.65 ± 0.15 nM; rat *KISS1R*: K_i_ = 2.04 ± 0.03 nM	Kotani et al., 2001 [1]
	CHO-K1 cells expressing human or rat *KISS1R*	10 pM–1 µM	Aequorin-based functional assay to measure Ca^2+^	Human *KISS1R*: EC50 = 7.22 ± 0.07 nM; rat *KISS1R*: EC50 = 1.33 ± 0.01 nM	Kotani et al., 2001 [1]
KP-13	CHO-K1 cells expressing human or rat *KISS1R*	10 pM–1 µM	Competitive binding assay	Human *KISS1R*: K_i_ = 4.23 ± 0.10 nM; rat *KISS1R*: K_i_ = 2.08 ± 0.04 nM	Kotani et al., 2001 [1]
	CHO-K1 cells expressing human or rat *KISS1R*	10 pM–1 µM	Aequorin-based functional assay to measure Ca^2+^	Human *KISS1R*: EC50= 4.62 ± 0.02 nM; rat *KISS1R*: EC50 = 1.38 ± 0.02 nM	Kotani et al., 2001 [1]
KP-10	CHO-K1 cells expressing human or rat *KISS1R*	10 pM–1 µM	Saturation binding assay	Human *KISS1R*: Kd = 1.9 ± 0.4 nM; rat *KISS1R*: Kd = 1.0 ± 0.1 nM	Kotani et al., 2001 [1]
	CHO-K1 cells expressing human or rat *KISS1R*	10 pM–1 µM	Competitive binding assay	Human *KISS1R*: K_i_ = 2.33 ± 0.13 nM; rat *KISS1R*, K_i_ = 1.59 ± 0.07 nM	Kotani et al., 2001 [1]
	CHO-K1 cells expressing human or rat *KISS1R*	10 pM–1 µM	Aequorin-based functional assay to measure Ca^2+^	Human *KISS1R*: EC_50_= 4.13 ± 0.02 nM; rat *KISS1R*: EC_50_ = 1.17 ± 0.02 nM	Kotani et al., 2001 [1]
	Men and women	0.3–32 nmol/kg	LH and FSH	Elevated	Jayasena et al., 2011 [77]
FTM080	CHO cells expressing *KISS1R*	NA	Ca^2+^ assay	EC_50_ = 0.45–0.69 nM	Tomita et al., 2007 [78]; Tomita et al., 2008 [79]
	CHO cells expressing *KISS1R*	NA	Competitive binding assay	IC_50_ = 0.71 nM	Tomita et al., 2008 [79]
	Anestrous ewe	500–5000 pmol/kg BW	LH	Elevated	Whitlock et al., 2015 [80]
KISS1-305	Male rats	1–4 nmol/h	LH, testosterone, gene expression, and genital organ	Elevated plasma LH and testosterone; no alteration in *gnrh* expression; reduced genital organ weight.	Matsui et al., 2012 [81]
	CHO cells expressing human *KISS1R*	NA	Ca^2+^ assay	EC_50_ = 4.8 nM	Asami et al., 2013 [55]
	CHO cells expressing human *KISS1R*	NA	Cell membrane binding assay	Human *KISS1R*: K_i_ = 0.089 nM; rat *KISS1R*: K_i_ = 0.10 nM	Asami et al., 2013 [55]
TAK-448/MVT-602	Male rats	0.1 nmol/h	LH/FSH/testosterone/GnRH level and gene expression	Decreased plasma LH, FSH, testosterone, and hypothalamic GnRH	Matsui et al., 2012 [81]
	CHO cells expressing rat *KISS1R*	NA	Ca^2+^ assay	EC_50_ = 632 pM	Matsui et al., 2014 [82]
	CHO cells expressing rat *KISS1R*	NA	Competitive binding assay	IC_50_ = 460 pM	Matsui et al., 2014 [82]
	Male rats	0.008–8 µmol/kg/day	LH, testosterone, and genital organ weights	Elevated plasma LH and testosterone; reduced genital organ weights	Matsui et al., 2014 [82]
	Healthy men/patients with prostate cancer	0.01–6 mg/day	Testosterone level	Healthy men: increased; patient: decreased	MacLean et al., 2014 [83]
	CHO cells expressing human or rat *KISS1R*	NA	Ca^2+^ assay	Human *KISS1R*: EC_50_ = 5.2 nM; rat *KISS1R*: EC_50_ = 36 nM	Nishizawa et al., 2016 [84]
	HEK293 cells expressing FLAG-*KISS1R*	10 pM–1 µM	IP1 assay	EC_50_ = 10.71	Abbara et al., 2020 [85]
	Women	0.01/0.03 nmol/kg	LH	Elevated	Abbara et al., 2020 [85]
TAK-683	Healthy men	0.01–2.0 mg/day	LH, FSH	Suppressed LH, FSH, and testosterone	Scott et al., 2013 [86]
	CHO cells expressing human or rat *KISS1R*	NA	Ca^2+^ assay	Human *KISS1R*: EC_50_ = 0.33 nM; rat *KISS1R*: EC_50_ = 1.3 nM	Asami et al., 2014 [87]
	CHO cells expressing human *KISS1R*	NA	Cell membrane binding assay	Human *KISS1R*: K_i_ = 0.036 nM; rat *KISS1R*: K_i_ = 0.069 nM	Asami et al., 2014 [87]
	CHO cells expressing rat *KISS1R*	NA	Ca^2+^ assay	EC_50_ = 180 pM	Matsui et al., 2014 [82]
	CHO cells expressing rat *KISS1R*	NA	Competitive binding assay	IC_50_ = 170 pM	Matsui et al., 2014 [82]
	Male rats	0.008–8 µmol/kg/day	LH, testosterone, and genital organ weights	Elevated plasma LH and testosterone; reduced genital organ weights	Matsui et al., 2014 [82]
RF9	Male and female rats	0.01–20 nM	LH and FSH	Evoked a dose-dependent increase in LH and FSH levels	Pineda et al., 2010 [88]
	Ewes	2.1–18.6 μmol/h per ewe	LH	Induced plasma LH	Caraty et al., 2012 [89]
	*GnRH*-GFP or *Kiss1r*^-^ null male and female rats	0.05, 0.2, and 1μM	Cell-attached voltage of GnRH neuron	Generated an inward current in GnRH neurons	Liu et al., 2014 [90]
	CHO cells expressing human *KISS1R*	10 pM–100 µM	Binding assay	Kd = 16 µM	Min et al., 2015 [91]
	CHO cells expressing human *KISS1R*	1 nM–100 µM	Ca^2+^ assay	EC_50_ = 3 µM	Min et al., 2015 [91]
	CHO cells expressing human *KISS1R*	1 nM–10 µM	IP1	EC_50_ = 0.16 µM	Min et al., 2015 [91]
	*NPFFR1*^−/−^, *KISS1R*^−/−^, and *NPFFR1*^−/−^/*KISS1R*^−/−^ mice	5 nM/5 µL	LH	Stimulated a robust LH increase in Npffr1^−/−^ mice	Min et al., 2015 [91]
Musk ambrette	HEK293 cells expressing human *KISS1R*	1 nM–66 µM	Ca^2+^ assay	EC_50_ = 16.71 µM	Yang et al., 2024 [25]
	HEK293 cells expressing human *KISS1R*	2 nM–115 µM	pERK assay	EC_50_ = 55.86 µM	Yang et al., 2024 [25]
	Murine hypothalamic cells	6.25–50 µM	*Gnrh1* expression	EC_50_ = 21.94 µM	Yang et al., 2024 [25]
	*GnRH3*-GFP zebrafish	0.1–1 µg/mL	*Gnrh3* expression	Expanded GnRH neuronal area	Yang et al., 2024 [25]

NA, not available.
